# National water shortage for low to high environmental flow protection

**DOI:** 10.1038/s41598-022-06978-y

**Published:** 2022-02-22

**Authors:** Davy Vanham, Lorenzo Alfieri, Luc Feyen

**Affiliations:** 1 European Commission, Joint Research Centre (JRC), Ispra, Italy; 2 CIMA Research Foundation, Savona, Italy

**Keywords:** Hydrology, Sustainability

## Abstract

Global freshwater biodiversity has been decreasing rapidly, requiring the restoration and maintenance of environmental flows (EFs) in streams and rivers. EFs provide many ecosystem services that benefit humans. Reserving such EFs for aquatic ecosystems, implies less renewable water availability for direct human water use such as agriculture, industry, cities and energy. Here we show that, depending on the level of EF protection, global annual renewable water availability for humans decreases between 41 and 80% compared to when not reserving EFs. With low EF protection, currently 53 countries experience different levels of water shortage, which increases to 101 countries for high EF protection. Countries will carefully have to balance the amount of water allocated to humans and the environment.

## Introduction

Globally, monitored population sizes of mammals, fish, birds, reptiles and amphibians have declined by 68% on average between 1970 and 2016^1^. Freshwater species have been disproportionally impacted and decreased by 84%. Almost one in three freshwater species is threatened with extinction^1^, which is consistently higher than for their terrestrial counterparts^2^. The violation of environmental flows (EFs) due to the water footprint of humanity^3–5^ is a major reason for this rapid decline in aquatic biodiversity^6^. Such EFs are required to maintain ecosystem integrity in streams, rivers, wetlands, riparian zones and estuaries. EFs also provide many additional ecosystem services, with direct links to specific Sustainable Development Goals (SDGs)^7,8^. As an example, EFs sustain fish stocks and other aquatic life, which contribute as nutrition biomass directly to SDG 2 “zero hunger”. In the Mekong river basin, the second most aquatic biodiverse river basin in the world^9^, freshwater fish biomass contributes the bulk of animal protein intake. A dish with fish and rice therefore requires EFs as well as enough water for agriculture. Restoring and maintaining EFs is thus essential for humanity^10^.

Although locally, empirical quantitative relationships between various degrees of flow alteration and ecological responses have been derived, EFs are still unknown for the vast majority of freshwater and estuarine ecosystems^11^. With over 200 existing EFs methods^10^, it is a challenge to quantify how much water should be reserved to sustain ecosystems and how much water is available for direct human use such as agriculture, industry, cities and energy.

Here we assess national per capita renewable water availability worldwide, accounting for high respectively low aquatic ecosystem protection by means of two well established EF methods. As a measure for high ecosystem protection, we use the presumptive standard for EFs by Richter et al.^11^, which attributes 80% of natural monthly river flows as EF (EF_PROT_). As a measure representative for minimum flow recommendations (EF_MIN_), we use the monthly Q_95_, that is, the flow exceeded for 95 per cent of each month. We then substract EFs from national renewable available water and identify the corresponding levels of human water shortage for each country. Both EF_PROT_^4,12–19^ and EF_MIN_^18,20–25^ are widely used in different water management studies.

We compute renewable water availability in high spatial and temporal detail, with the established hydrological open-access model Lisflood^26^. Natural or pristine water availability is water availability without human interventions, such as water use or water infrastructure (dams, pipes, …). The model works at a spatial resolution of 0.1 degrees (11.1 km at equator), with a daily time step for the period 1980–2018, and generates natural water availability as the sum of renewable surface and groundwater.

We aggregate daily simulated water resources to monthly values in order to account for the intra-annual variability in water availability and EFs, and compute the resulting per capita water availability and shortage on an annual level. We distinguish between natural or pristine water availability, referred to as renewable water availability without EF protection (WA_noEF, Table 1), human renewable water availability with low EF protection (WA_EF_MIN_ = WA_noEF − EF_MIN_) and human renewable water availability with high EF protection (WA_EF_PROT_ = WA_noEF − EF_PROT_).

**Table 1:** Different water balance components used in this study.

Component	Abbreviation	Description
renewable water availability without EF protection	WA_noEF	Natural or pristine water availability
EF with low ecosystem protection	EF_MIN_	As measure representative for minimum flow recommendations, we use the monthly Q_95_, that is, the flow exceeded for 95 per cent of each month
human renewable water availability with low EF protection	WA_EF_MIN_	WA_noEF − EF_MIN_
EF with high ecosystem protection	EF_PROT_	Presumptive standard for EFs by Richter et al.^11^, which attributes 80% of natural monthly river flows as EF
human renewable water availability with high EF protection	WA_EF_PROT_	WA_noEF − EF_PROT_
Internal WA		Internally produced renewable water availability
Total WA		To compute annual national total renewable water availability, we add inflow to internal amounts

Water shortage estimations are derived by combining average annual country renewable water availability estimates with population statistics. Based on the corresponding per capita water availability, different water shortage classes are defined for each country (Table 2). Following the widely used Falkenmark indicator^27^, the threshold between water shortage and no water shortage is set at 1700 m^3^ per person per year. Below that threshold, there are five water shortage classes ranging from 1 (chronic water shortage) to 5 (absolute water shortage). Above that threshold, two classes without water shortage are defined.

**Table 2:** Different renewable water availability classes, identifying water shortage.

Class	m^3^ per person per year	Liters per person per day	Description, with indication of data source
No water shortage: highest water availability	> 5000	> 13,699	Above the threshold of 1700 m^3^ identified by Falkenmark et al.^27^, with an additional classification between high and highest water availability
No water shortage: high water availability	1700–5000	4658–13,699	
Water shortage: class 1 (chronic water shortage)	1000–1700	2740–4658	The threshold 1700 m^3^ was identified by Falkenmark et al.^27^ as limit under which a country becomes water stressed
Water shortage: class 2 (high water shortage)	500–1000	1370–2740	According to Falkenmark et al.^27^, below 1000 m^3^ a country is said to be experiencing water scarcity
Water shortage: class 3 (very high water shortage)	100–500	274–1370	According to Falkenmark et al.^27^, below 500 m^3^ a country is said to be experiencing absolute water scarcity. 500 m^3^ equaled the lowest need in a modern semi-arid country (Israel)
Water shortage: class 4 (extreme water shortage)	18.3–100	50–274	100 m^3^ per year or 274 L per day is chosen as a proxy for the requirement for modern municipal water use. This value approximates the current municipal water use of the UK, Israel, Singapore^32^ and Hong Kong^75^
Water shortage: class 5 (absolute water shortage)	< 18.3	< 50	18.3 m^3^ per year or 50 L per day as minimum domestic water requirement^29,76^. This value is composed of 3 L for drinking, 20 L for sanitation (flushing toilets), 15 L for bathing and 10 L for cooking food and cleaning dishes

National per capita water availability and potential water shortage are quantified for the year 2020, both as internally produced water availability and total water availability when water enters a country through inflow from upstream river basins. We use the transboundary river basins database to address the contribution of this externally produced water availability^28^.

We use water shortage as indicator. There are two main physical water scarcity indicators: water shortage and water stress^8^. We do not account for economic water scarcity^8^.

- Water shortage measures water availability per person. Given a certain water endowment and per capita water requirement, water shortage can therefore be seen as population-driven scarcity. The Falkenmark indicator^27^ is a water shortage indicator.
- Water stress measures water use relative to water availability. Water stress can be seen as demand-driven scarcity, potentially occurring even when population is low, for instance because of large water-use for producing agricultural or industrial products for populations elsewhere. SDG indicator 6.4.2 is a water stress indicator

## Results

**Figure 1:**
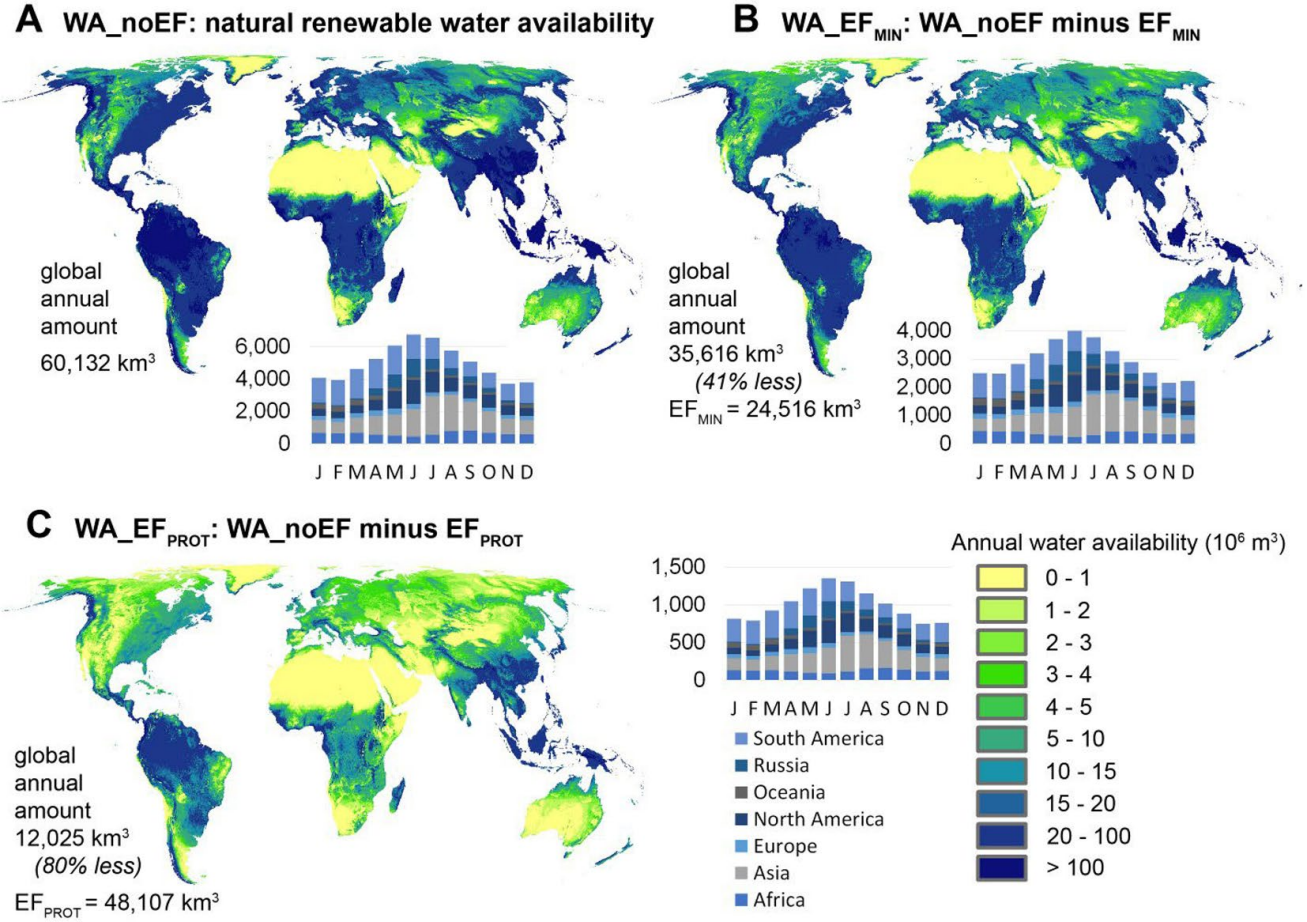
Global renewable water availability in high spatial resolution (0.1 degrees), without and with accounting for Efs. (**A**) map of global annual WA_noEF (in 10^6^ m^3^) and graph with monthly WA_noEF (in km^3^) per continent; (**B**) map of global annual WA_EF_MIN_ (in 10^6^ m^3^) and graph with monthly WA_EF_MIN_ (in km^3^) per continent and (**C**) map of global annual WA_EF_PROT_ (in 10^6^ m^3^) and graph with monthly WA_EF_PROT_ (in km^3^) per continent. Maps generated with ArcMap (Version 10.8, https://www.esri.com).

Estimated global annual natural renewable water availability (WA_noEF) amounts to 60,132 km^3^ (or 7669 m^3^ per person per year), with high temporal and spatial variation in availability spread over the globe (Fig. 1). Monthly WA_noEF amounts peak in June and July, driven by the monsoon in Asia, whereas the winter months of the Northern Hemisphere show the lowest WA_noEF amounts. Ensuring minimum monthly EFs throughout the world adds up to a global annual EF_MIN_ amount of 24,516 km^3^. This leaves 35,616 km^3^ (WA_EF_MIN_) for direct human use, so 41% less as compared to WA_noEF. Ensuring high monthly EFs adds up to a global annual EF_PROT_ amount of 48,107 km^3^, leaving 12,025 km^3^ (WA_EF_PROT_) for direct human use. This is 80% less as compared to WA_noEF.

Whereas the proportions 80% for EF_PROT_ and 20% for WA_EF_PROT_ related to WA_noEF are spatially homogeneous, there are wide spatial variations in the proportions of EF_MIN_ and WA_EF_MIN_ related to WA_noEF (Figure S1). For EF_MIN_ as proportion of WA_noEF (Figure S1A), the mean of all grid cells is 26% (SD 19%). For WA_EF_MIN_ as proportion of WA (Figure S1B)_noEF, the mean of all grid cells is 74% (SD 19%).

**Figure 2:**
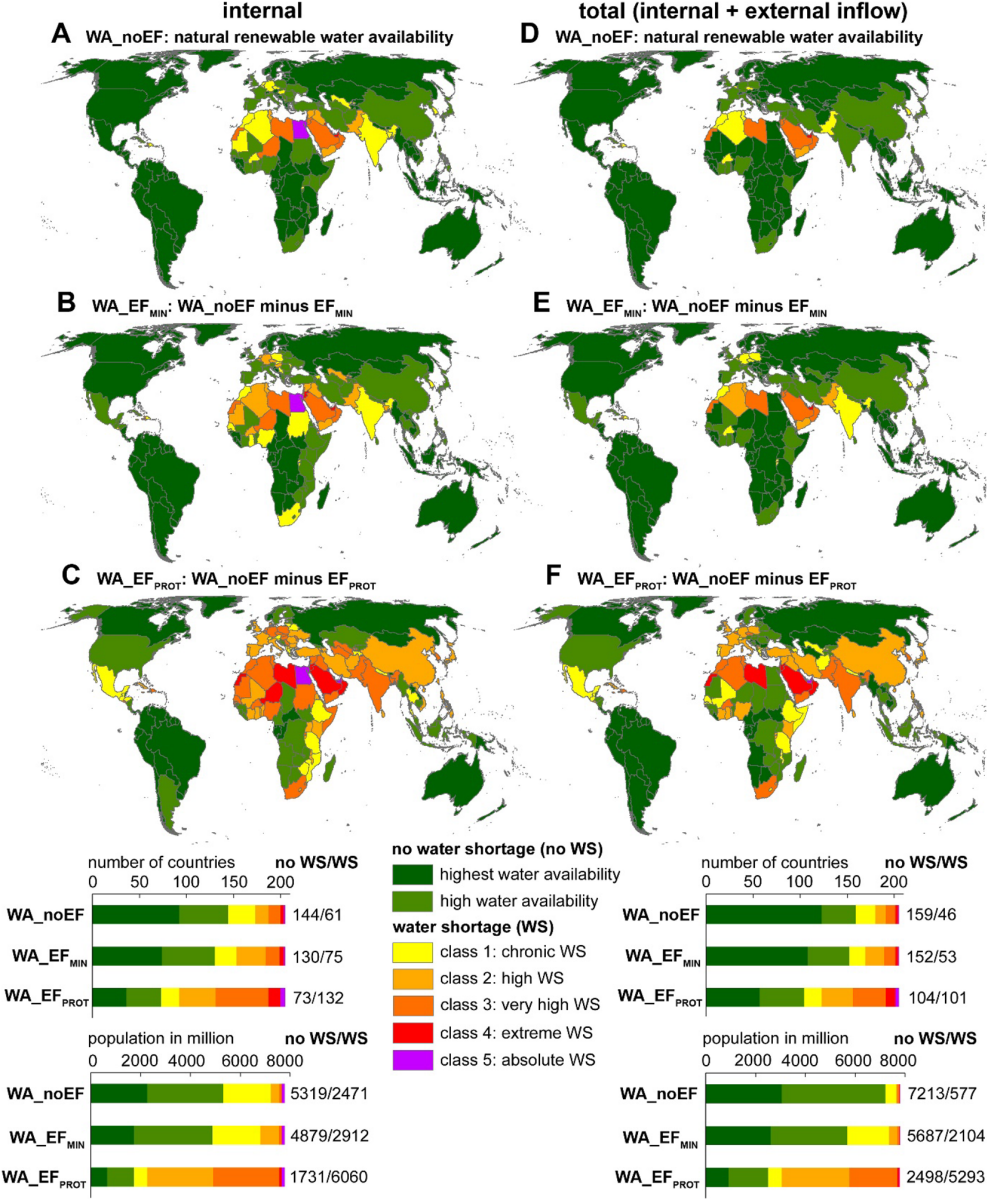
National internal (left column) and total (right column) renewable water shortage for WA_noEF (**A**, **D**), WA_EF_MIN_ (**B**, **E**) and WA_EF_PROT_ (**C**, **F**), year 2020. The bottom graph shows the number of countries and associated population under different levels of water shortage. Maps generated with ArcMap (Version 10.8, https://www.esri.com).

For the population status in the year 2020, internal and total water shortage levels differ substantially among the 205 countries and between the different levels of EF protection (Fig. 2 and Database S1). When only accounting for internal renewable water availability, neglecting EFs results in 144 countries (with a population of 5319 million) without water shortage and 61 countries (with a population of 2471 million) with water shortage. Ensuring minimum EFs (EF_MIN_), results in 130 countries (with a population of 4879 million) without water shortage and 75 countries (with a population of 2912 million) with water shortage. Ensuring high EFs (EF_PROT_), results in 73 countries (with a population of 1731 million) without water shortage and 132 countries (with a population of 6060 million) with water shortage. There is a substantial increase in the amount of countries and population under water shortage when ensuring EF_PROT_ instead of EF_MIN_, with populous countries such as China, Mexico or Iran moving to water shortage.

The amount of countries with very high to absolute internal water shortage (classes 3 to 5) increases from 18 (with a population of 224 million) for WA_noEF to 21 (with a population of 235 million) for WA_EF_MIN_ to 74 (with a population of 2881 million) for WA_EF_PROT_ (Fig. 2). Under EF_PROT_, India adds a large population to class 3. Egypt and Bahrain are already for WA_noEF internally under class 5 water shortage, with Qatar, the United Arab Emirates and Kuwait moving from class 4 for WA_noEF to class 5 for WA_EF_PROT_. Jordan moves from class 3 for WA_noEF to class 4 for WA_EF_MIN_. Countries that shift from class 3 for WA_noEF to class 4 for WA_EF_PROT_ are different Middle eastern and African countries (Palestina, Israel, Oman, Saudi Arabia, Western Sahara, Niger and Lybia) as well as island states (Malta, Singapore, Barbados, Aruba and Mayotte). Many island states in the Caribbean as well as Cyprus, which do not have inflow from abroad, shift to class 3 for WA_EF_PROT_.

When accounting for water inflows from upstream countries, renewable water availability in different countries increases and the amount of countries as well as number of people under water shortage decrease (Fig. 2). Not accounting for EFs results in 159 countries (with a population of 7213 million) without water shortage and 46 countries (with a population of 577 million) with water shortage. Ensuring minimum EFs (EF_MIN_) results in 152 countries (with a population of 5687 million) without water shortage and 53 countries (with a population of 2104 million) with water shortage. Ensuring high EFs (EF_PROT_) results in 104 countries (with a population of 2498 million) without water shortage and 101 countries (with a population of 5293 million) with water shortage.

Inflow from abroad changes the water shortage situation of some countries dramatically. Most notably, the inflow from the Nile shifts Egypt from class 5 water shortage to no water shortage (WA_EF_PROT_ = 2099 m^3^ per person per year), as 99% of total water availability comes from abroad (Figure S2). Other countries with high populations that shift from water shortage to no water shortage even when ensuring high EFs (EF_PROT_) include Bangladesh (87% from abroad), Thailand (52–57% from abroad), Romania (74–77% from abroad), Ukraine (66–67% from abroad), Sudan (82–84% from abroad), Zimbabwe (67–71% from abroad) and Mozambique (69% from abroad).

When accounting for total renewable water availability (Fig. 2), the amount of countries with very high to absolute internal water shortage (classes 3 to 5) increases from 14 (with a population of 92 million) for WA_noEF to 16 (with a population of 92 million) for WA_EF_MIN_ to 49 (with a population of 2016 million) for WA_EF_PROT_ (Fig. 2). Under EF_PROT,_ India, Pakistan and South Africa add large populations to class 3. Countries with at least class 3 water shortage under EF_PROT_ are located in South and East Asia (India, Pakistan, South Korea), the Middle East (Bahrain, Qatar, United Arab Emirates, Kuwait, Jordan, Palestina, Israel, Oman, Saudi Arabia, Yemen, Lebanon), North and Southern Africa (Morocco, Libya, Tunisia, Algeria, Burkina Faso, Western Sahara, South Africa), Europe (Belgium, Czech Republic, Kosovo) as well as many island states spread over the world.

## Discussion

We compute a first-time detailed national population-driven water shortage analysis under no, low and high EF protection, by means of a state-of-the-art global hydrological model. We base our analysis on estimated monthly water availability and EF amounts, data we provide for each country in Database S1. We observe that reserving different levels of EFs has a high impact on national annual per capita water availability for human use as well as resulting water shortage levels. For the year 2020, ensuring the presumptive standard of high environmental flow protection (EF_PROT_), results in 101 countries (with a population of 5293 million) experiencing different levels of water shortage, of which 49 (with a population of 2016 million) with very high to absolute internal water shortage (classes 3 to 5). But even low EF protection (EF_MIN_) results in 53 countries (with a population of 2104 million) with water shortage, of which 16 (with a population of 92 million) with extreme to absolute internal water shortage.

The population-driven water scarcity metric we use, is strongly based on the Falkenmark indicator^27^, a well-established and widely used indicator^8,29–31^. Reasons for the wide acceptance of this indicator are multiple: it is simple and intuitive, and data on human population are readily available.

Also, although water should be properly managed within river basins, even when they cross international borders, most countries are primarily concerned about water availability within national borders. Our assessment provides such water availability estimations and has shown that several countries are highly dependent on inflow from abroad. This clearly shows the necessity for bringing together different sectors and stakeholders at all scales from local to transboundary in river basin management. SDG 6 therefore has a dedicated target 6.5 which aims at: “*By 2030, implement integrated water resources management at all levels, including through transboundary cooperation as appropriate*”.

Our indicator does not grasp efforts of countries to reduce their actual water abstraction. Israel, representing with 500 m^3^ per person a proxy for the lowest need in a modern semi-arid country^27^ (the cut-off for water shortage class 3), has in recent times further reduced its total per capita water abstraction to less than 300 m^3^ per year^32^, by implementing measures such as highly efficient irrigation, water recycling and desalination. It has thereby surpassed its peak water demand, decoupling GDP from water abstraction, in the same way many developed nations including the USA have^33,34^. According to Gleick^33^, such a decoupling of water withdrawals from population and economic growth has occurred in the USA due to three key reasons. First, substantial technological improvements reduced the amount of water required to meet urban, industrial, and agricultural needs. Second, changes in the overall structure of the US economy have also played a role in this transition, including a shift in water-intensive manufacturing to overseas locations. Third, a change away from water-intensive once-through cooling systems for thermal power plants has reduced the amount of water required to produce a unit of energy.

The threshold value for class 5 (minimum domestic water demand) is a universal amount. The threshold value for class 4 (municipal water demand) is a realistic target value which can be achieved in a developed country. The threshold values for water shortage classes 1 to 3 are dependent on climatological conditions and are to be regarded as indicative, not absolute.

Our assessment does not address differences in water demands between countries or intranational differences^35^ in water availability. Another indicator on water scarcity, i.e. water stress, the ratio between water use and water availability, can grasp such information^4,8,30^, but is not in the scope of our study. Nor does our study give any indication on the status of national water supply infrastructure (e.g. water pipes, water diversions, reservoirs etc.). Our study should therefore be seen a first-pass assessment that gives indications on national water shortage, to be examined in detail on country level. In addition, we do not account for the impact of climate change on future water shortage.

Countries will carefully have to balance how much water they attribute as EFs and how much for direct human use. Which EFs are eventually optimal in specific locations requires further investigation of environmental needs through sophisticated EF assessment methodologies, such as habitat simulation methods and holistic methods, supported by local field measurements campaigns. We expect such local EF flows to be situated within the range between EF_PROT_ and EF_MIN_ we have determined in our analysis.

Human water use will have to be managed and optimized^36^, surpassing peak per capita demands, by increasing water efficiency and productivity^37–41^, water recycling, addressing the groundwater component of EFs^42^, holistic river basin management and end-of-supply-chain interventions such as food waste reduction, dietary shifts and choice of energy sources^19,43–47^. In addition, river fragmentation by infrastructure such as dams and weirs will have to be managed and river connectivity ensured in order to rehabilitate and maintain EFs^48–50^, to bend the curve on global aquatic biodiversity loss^6^ and to ensure the multiple ecosystem services provided to humans by EFs. The EU, e.g., has in its biodiversity strategy for 2030^51^, targeted that at least 25,000 km of rivers will be restored into free-flowing rivers by 2030, through the removal of primarily obsolete barriers and the restoration of floodplains and wetlands. To bend the curve on global aquatic biodiversity loss, in addition, good water quality and habitats need to be restored^52–54^, global warming limited^55^ and overexploitation^56^ and invasive species^57^ tackled. Overall, many different actions need to be undertaken to speed progress towards SDG 6^36^, addressing win–win situations and trade-offs within the water-energy-food-ecosystem (WEFE) nexus^58,59^. This requires the inclusion of many different actors at different scales^60^.

## Materials and methods

### Computing (monthly) natural water availability with the Lisflood model

To compute natural water availability, we used the open-source distributed semi-physically based hydrological model Lisflood^26^, a well established model used in different studies from local to global level^18,61–63^. Lisflood accounts for rainfall-runoff-routing in the river network, as well as several surface and sub-surface hydrological processes, including plant interception, evapotranspiration, soil freezing, snow accumulation and melting, surface runoff, lakes and reservoirs, water abstraction, infiltration, preferential flow, redistribution of soil moisture within the soil profile, drainage to the groundwater system, groundwater storage, and base flow. Surface runoff is produced at every grid cell and routed through the river network using a kinematic wave approach^64^.

High-quality spatial datasets in hydrological modeling are crucial to avoid over‐parameterization and reduce the dimensionality of the model calibration. Spatial datasets used in Lisflood include:

- topography maps (digital elevation model, local drainage direction, slope gradient, elevation range). We use the Shuttle Radar Topography Mission (SRTM)^65^ for elevation and the global river network database^66^ for river network and flow direction
- soil (soil texture classes, soil depth). We use SoilGrids1km^67^ for soil information
- land use (land use classes, forest fraction, fraction of urban area). We use GlobCover 2009^68^ for land use and the SPOT-VGT data^69^ for monthly maps of Leaf Area Index.
- channel geometry (roughness coefficient, bankfull channel depth, channel gradient, length, bottom width, and side slope). We use the Global Width Database of Large Rivers^70^ for river widths

We used the Lisflood setup and parameterization of the GloFAS-Reanalysis v3.0^62^, a state-of-the-art global streamflow reanalysis with median scores at 1226 calibration stations within 66 world countries of Kling-Gupta Efficiency KGE = 0.67 and correlation r = 0.8. All atmospheric variables to run the model were extracted from the ERA5^71^ reanalysis and regridded from the original resolution of 31 km to the model resolution (~ 11 km) using nearest neighbour interpolation. Lisflood requires, as input, near surface air temperature, precipitation and potential evapotranspiration. The latter was estimated with the Penman–Monteith equation as described in Supit et al.^72^, using daily average temperature, wind speed, relative humidity and solar radiation as input.

We run a 40 year simulation for 1980–2018, excluding all the human influence, i.e., without the effect of reservoirs and of all water abstractions. Resulting daily discharges of natural or pristine flows were aggregated at monthly resolution. The simulated discharges incorporate surface water flows as well as base flows (renewable groundwater), resulting in total renewable water availability.

As a result, we calculate a global annual renewable WA_noEF of 60,132 km^3^ as average for the period 1980–2018. Past assessments found a range of WA_noEF amounts, depending on modelling methodology, time period, inclusion of anthropogenic water use as well as inclusion of Greenland or not^73^. LISFLOOD is on the higher end of the multi-model range of water availability computations^73^. A recent assessment for the period 1970–2005^15^ quantified for three models an average WA_noEF of 54,100 km^3^, a value close to our own estimate here.

### Environmental flows (EFs)

We use two well-established hydrological EF methodologies to account for minimum and maximum EF protection:

- EF_PROT_: the presumptive standard for EFs by Richter et al.^11^, which defines 80% of the (monthly) natural flow as EF; the remaining 20% is considered as water available for human use.
- EF_MIN_: the monthly Q_95_ (the flow exceeded for 95 per cent of the month). The Q_95_ is often used as low flow indices

These EFs are calculated with a daily time step. Both selected methods are hydrological methods. EF methods can be classified into four types: hydrological methods; hydraulic rating methods; habitat simulation methods; and holistic methods. Latter methods require large amounts of data which are not available at the global level. As such, we choose only hydrological methods.

### National internal and total renewable water availability

We assess annual national internal renewable water availability as the sum of monthly water availability, for the nations and geographical entities and related population numbers in the year 2020 as listed by the UN^74^. We distinguish between:

- renewable water availability without EF consideration (WA_noEF)
- human renewable water availability water with low EF protection (WA_EF_MIN_ = WA_noEF − EF_MIN_)
- human renewable water availability water with high EF protection (WA_EF_PROT_ = WA_noEF − EF_PROT_).

Thresholds between different per capita water shortage classes are defined as listed in Table 2.

To compute annual national total renewable water availability, we add inflow to internal amounts. The inflow is determined by means of the inflow from upstream river basins, for which we use the transboundary river basins database^28^ (Figure S2). As rules, we determine that all inflow from upstream (upstream WA_noEF, WA_EF_MIN_ or WA_EF_PROT_) adds to the internal water availability (internal WA_noEF, WA_EF_MIN_ or WA_EF_PROT_) of a country. When a river forms the border between two countries, we assume that this flow is fully available to both countries (only for the upstream basin part, upto where the river stops forming an international border). As such, we compute a maximal total renewable water availability. These values are of course theoretical and represent an overestimation. In reality, upstream countries alter (monthly) water availability for downstream countries by means of water storage infrastructure such as dams as well a consumptive water use^8^.

## Supplementary Information

Supplementary Information 1.

Supplementary Information 2.

## Data Availability

Most data are available in the main text or the supplementary materials. Additional data such as the monthly georasters of EFs and renewable water availability are available upon reasonable request with the corresponding author. Lisflood is an open source model.
