# Supplementary material for: National water shortage for low to high environmental flow protection
Source: Sci Rep. 2022 Feb 22;12:3037. doi: 10.1038/s41598-022-06978-y (PMC8864015; doi:10.1038/s41598-022-06978-y)
Supplement: Supplementary file 1 — Supplementary Information 1. [file 41598_2022_6978_MOESM1_ESM.pdf]

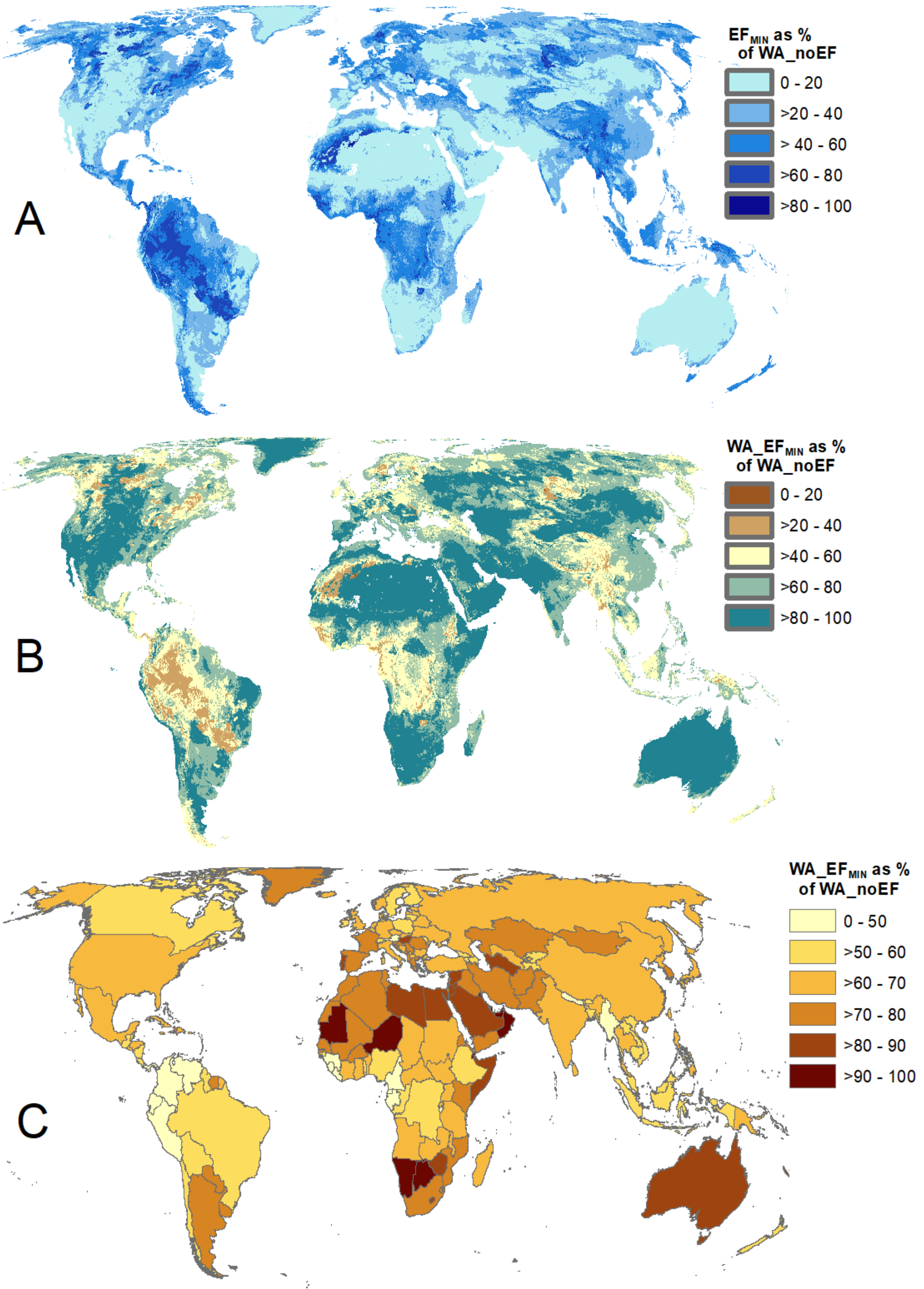

Fig S1. A) Grid level percentage EF_{MIN} to WA_noEF; B) Grid level percentage WA_EF_{MIN} to WA_noEF; B) national average percentage WA_EF_{MIN} to WA_noEF;

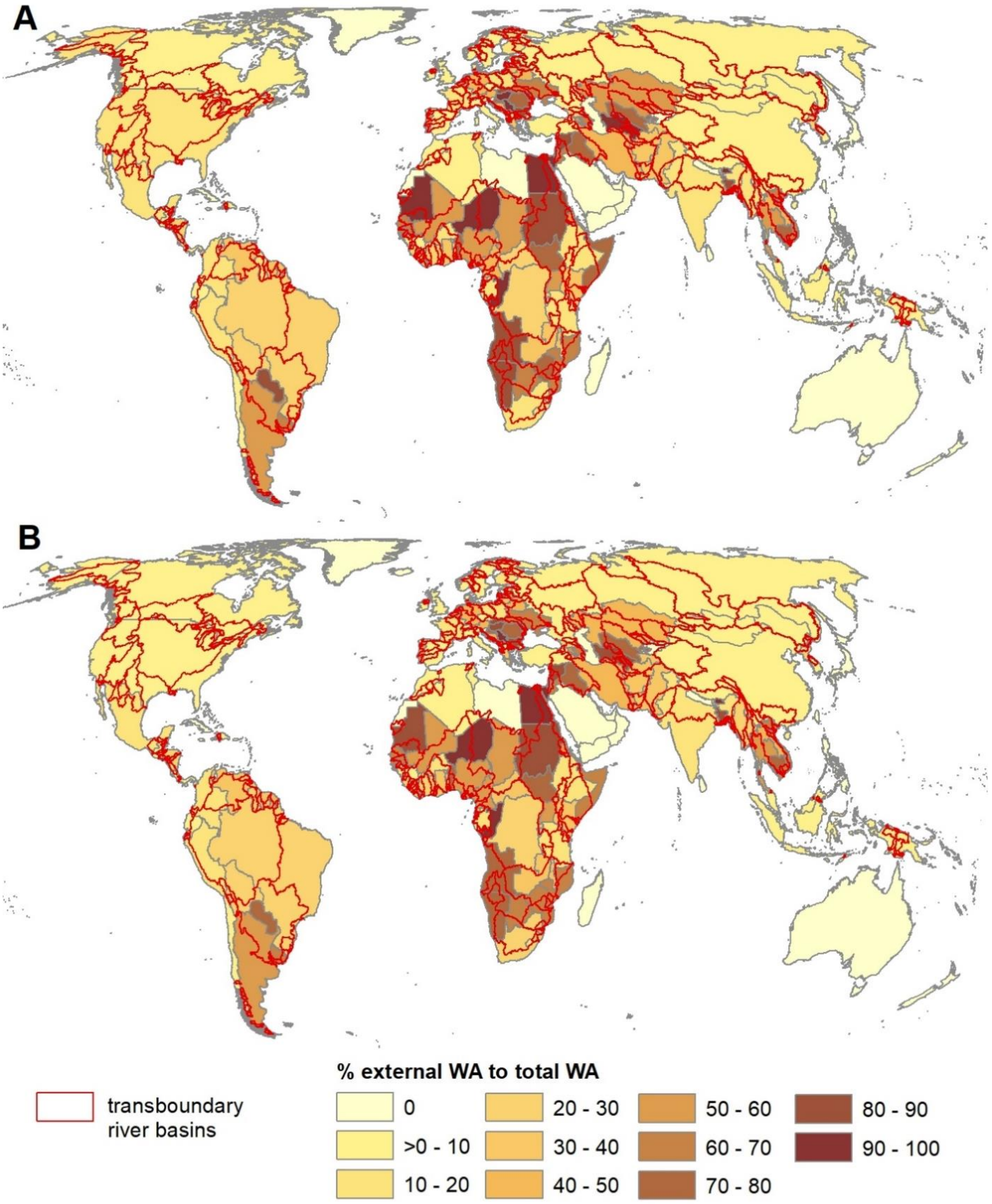

Fig. S2. Percentage national external water availability (WA) to total WA, for A) WA_noEF and WA_EF_{PROT} and B) WA_EF_{MIN}.
